# Predictors of personal depression stigma in medical students in China: differences in male and female groups

**DOI:** 10.1080/10872981.2022.2093427

**Published:** 2022-06-23

**Authors:** Lei Qiu, Yawen Feng, Jiaxin Luo, Yinuo Zhang, Qin Yang

**Affiliations:** a International School of Public Health and One Health, Hainan Medical University, Hainan, P. R. China; b School of Management, Hainan Medical University, Hainan, P. R. China; c Xiangyang Central Hospital, Affiliated Hospital of Hubei University of Arts and Science, Xiangyang, P. R. China

**Keywords:** Personal stigma, depression, gender differences, medical students, China

## Abstract

Depression is common worldwide, and stigmatizing attitudes toward depression have proved to be one of the major barriers to seeking professional help. The purpose of this study was to evaluate the level of personal depression stigma and identify its predictive factors among medical students in Hainan, China, as well as explore the gender difference. A total of 2,186 medical students were recruited using stratified random cluster sampling and interviewed by structured anonymous questionnaires. Personal stigma was measured by the standardized Depression Stigma Scale (DSS). Multivariate linear regression models were used to identify predictors of stigma, and the interactions between gender and each predictor were included to test its gender difference. The mean score on DSS Scale was 13.71 ± 5.35, with males significantly higher than females (14.85 vs 12.99, *P* < 0.0001). Compared to females, males were more likely to agree with ‘I would not vote for a class cadre if I knew they had been depressed’ and ‘I would not make friends with him if I knew he had been depressed’. Multivariate linear regression analysis revealed that males’ personal stigma was predicted by being only child (*ß* = 1.01, *P* = 0.0083), moderate-to-severe depression (*ß* = 1.12, *P* = 0.0302), and lower self-rated academic core competitiveness (Competitive: *ß* = 1.29, *P* = 0.0088, Not at all/Somewhat competitive: *ß* = 1.04, *P* = 0.0381), while females’ personal stigma was only associated with moderate-to-severe depression (*ß* = 1.75, *P* < 0.0001). Significant interactions were found between gender and self-rated academic core competitiveness. Stigmatizing attitudes toward depression were prevalent among Chinese medical students, especially male students. Gender differences were found in the predictors of stigma. Effective measures must be taken to reduce the stigma of mental health among Chinese medical students.

## Introduction

Depression is one of the most prevalent mental disorders, which contributes significantly to the global disease burden. The global prevalence of depression was estimated at 4.4%, with 322 million people suffering from depression worldwide according to the World Health Organization (WHO) [1]. Medical students are a high-risk group for depression. A meta-analysis of 183 studies reported that 27.2% of medical students had depression or depressive symptoms [2]. Depression among university students has many negative effects on their physical and mental health, such as academic burnout, sleep disturbances, leading to addictive behaviors and even suicide [3–5].

Depression is more likely to be stigmatized when compared with physical disease [6]. Stigma was originally been defined as ‘the situation of the individual who is disqualified from full social acceptance’ by Erving Goffman in 1963 [7]. Depression stigma reflects individual’s negative attitudes/behaviors toward depression [8]. Depression stigma is generally divided into personal stigma and perceived stigma. Personal depression stigma usually refers to the negative attitudes formed by the negative cognition and negative emotional experience of depression, while perceived depression stigma usually refers to the devaluation and rejection of the depressed person by others [9]. Recently, stigma toward depression has been widely reported. A community-based survey in China found that the proportion of personal depression stigma was as high as 53.0% [10].

An increasing body of evidence suggests that depression stigma is the significant barrier for individuals with depression to seek professional help [11–14]. Research among 1,312 adults demonstrated that stigmatizing beliefs about depression could reduce the probability of seeking help from professionals such as GPs, psychiatrists, and psychologists [15]. Even medical students hold a discriminatory attitude toward depression. Moreover, they were concerned that seeking help could adversely affect them in advancement committee and course evaluations [14]. A research showed that 23.2% of American medical students believed that seeking help for depression would make them feel less intelligent as a medical student [16]. Furthermore, students with depressive symptoms were more sensitive to the stigma from their classmates and teachers [17]. Under the negative influence of the stigma, they felt that their classmates and teachers would look at them with disapproval after knowing their behavior of seeking professional psychological services. However, the more fearful they are of being stigmatized, the more reluctant they are to seek psychological help, which in turn leads to more severe depression. This creates a vicious circle [18].

If medical students suffering from depression do not receive professional treatment before engaging in clinical work, it may lead to many adverse outcomes, and even affect the health and life of patients. For example, Pereira-Lima et al. reviewed have reviewed 11 studies and concluded that physicians with depression have a higher risk of medical errors [19]. Therefore, it is urgent to explore the depression stigma of medical students and its related influencing factors, so as to propose a ‘prescription’ for stigma.

At present, many studies have begun to explore the influencing factors of depression stigma in different populations. Studies have shown that sociodemographic characteristics such as age, gender, education level, and occupation may be associated with depression stigma [10,20–22]. Besides, poor family function and depression experience was found to increase the risk of depression stigma [10,23]. However, depression stigma in China remains understudied and under-recognized. Few studies have investigated the level of depression stigma and its influencing factors among Chinese medical students. In addition, gender differences in depression stigma among college students are largely unknown [24]. In this study, we hypothesize that male college students exhibited more stigmatization than females toward depression. Although China’s only child policy ended in 2015, the current only child population is still huge, and the psychosocial differences between the only child group and the none-only child group have captured attracted the attention of a multitude of scholars [25].

Therefore, the primary purposes of our work are as follows. First, investigate the extent of personal depression stigma in Chinese medical students; second, find out whether there was any potential difference in personal depression stigma between male and female groups, as well as only child and none-only child groups; third, explore risk factors of stigma in male and female groups, respectively and test its gender difference. The present study may provide evidence for targeted interventions to reduce depression stigma among college students.

## Method

### Ethics statement

Ethical approval for this study was provided by the Human Research Ethics Committee, Hainan Medical University, Haikou, China. Each participant was voluntary and provided written informed consent prior to participation in this study. The identity information of all participants was kept strictly confidential.

### Participants and sampling

This cross-sectional study was conducted in Hainan Province, China from 1 January 2021 through 31 May 2021. Hainan is located in the most southern end of China and consists of Hainan Island, Paracel Islands, Spratly Islands, and Zhongsha Islands. There are three medical schools in Hainan Province, including Hainan Medical University, Hainan Health Management College, and Hainan Health Vocational College. The participants were selected using stratified cluster sampling design. First, five majors were randomly selected from each of the above three medical schools. Due to the overlap between the majors selected by each school, a total of 11 majors were selected, including preventive medicine, medical laboratory technology, clinical medicine, nursing, anesthesia, imaging, stomatology, pharmaceutical management, and other medical majors. Second, one class was randomly selected from each grade of the selected major, and all students in the selected class were recruited as study participants. This survey was conducted with a class as a unit. The students of the selected classes were gathered in the classroom, and the questionnaires were distributed by the uniformly trained investigators. Participants filled in questionnaires by themselves without interference from others, then investigators returned the questionnaire on the spot. After all questionnaires are collected, two members of our team checked data completeness and entered the data using EpiData 4.6.0.0 software. Overall, 2,200 medical students were obtained from three schools, giving a response rate of 96.49%. Of the returned 2,200 questionnaires, 14 were discarded due to large amounts of missing data. Finally, data on 2,186 medical students were included for analysis.

### Measuring instruments

The items of the questionnaire included depression, stigma, as well as demographic and academic characteristics. Demographic data included age, gender, whether only child or not, academic characteristics included self-rated academic core competitiveness and self-rated academic performance level. Self-rated academic core competitiveness is mainly measured by the following question, ‘Do you think you have academic core competitiveness?’, and the answers are divided into five categories: coded 1 = *not at all competitive* to 5 = *extremely competitive*. Self-rated academic performance level is mainly measured by the following question, ‘How does the self-assessment academic performance compare with peers in the same major?’, and the answers are divided into five categories (far below average/somewhat below average/average/somewhat above average/far above average).

The personal depression stigma subscale of the standardized Depression Stigma Scale (DSS) was used in the present study. The DSS-Personal scale is comprised of nine items (e.g., ‘Depression is a sign of personal weakness’), which are scored on a 5-point Likert scale (0 = *strongly disagree* to 4 = *strongly agree*) [26]. The total scores (range 0–36) were computed by summing all item score with higher total scores indicating higher levels of personal depression stigma. DSS-Personal scale has been extensively used in the investigation of different populations [20,27]. The Chinese version of the scale was used in our study, which showed excellent psychometric properties [10,28]. The internal consistency in the present sample was 0.76.

Depressive symptom was measured using the nine-item scale of the Patient Health Questionnaire (PHQ-9), which was a widely useful tool to screen for depression and assess depression severity [29]. Each item is rated on a 4-point Likert Scale (0 = *not at all* to 3 = *nearly every day*). Participants were asked to rate the frequency with which they have experienced certain symptoms over the last 2 weeks (e.g., ‘Little interest or pleasure in doing things’). Total score of this scale ranges from 0 to 27. Validated cutoff score was used in our analysis, with a total scores of 0–9 representing none to mild depression, and 10–27 representing moderate-to-severe depression [30]. The Cronbach’s alpha of the PHQ-9 in this study was 0.87.

### Statistical analysis

Statistical analysis was conducted using the Statistical Analysis System (SAS) 9.4 for Windows (SAS Institute Inc., Cary, NC, USA). First, we performed descriptive analyses on demographic characteristics of participants and reported numerical variables with mean and standard deviation (SD), while categorical data were reported as frequency and percentages. Second, the options of ‘agree’ and ‘strongly agree’ in DSS-Personal scale were combined into one option, and percentage frequencies and 95% CI were reported for each item. Pearson’s Chi-square test was used to assess the significant difference in each item on the DSS-Personal scale among different demographic variables (gender and whether only child) in the proportion of agreement. In addition, mean and SD were reported for total scores of DSS-Personal scale, and person correlation analysis was applied to assess the difference between male and female groups, as well as only child and none-only child groups. Finally, three multivariate linear regression models were used to explore the associated factors of personal stigma. Model 1 and model 2 analyzed female and male, respectively. Interactions between gender and each associated factor were included in Model 3 to assess any potential gender differences. All comparisons were two-tailed. The significance threshold was set at *P* = 0.05.

## Results

The participants’ demographic characteristics and the prevalence of depressive symptom are shown in Table 1. The mean age of the participants was 24.08 (SD = 1.70) years and 61.49% were female. More than two-thirds of the participants (67.19%) lived with at least one sibling. The proportion of participants with extremely/very competitive, competitive, not at all/somewhat competitive self-rated academic core competitiveness was 31.21%, 43.35%, and 25.44%, respectively. Most students (78.74%) thought their academic performance was at the average level or above. A total of 1,829 (83.67%) students reported none or minimal or mild depressive symptoms, while 357 (16.33%) reported moderate-to-severe depressive symptoms.

**Table 1.** Characteristics of participants.

Participant characteristics	*N* = 2,186	
	*n*	%
**Age (Years)** (Missing = 10)	24.08 ± 1.70	
**Gender** (Missing = 10)		
Male	838	38.51
Female	1338	61.49
**Whether only-child** (Missing = 4)		
Yes	716	32.81
No	1466	67.19
**Self-rated academic core competitiveness** (Missing = 4)		
Extremely/Very competitive	681	31.21
Competitive	946	43.35
Not at all/Somewhat competitive	555	25.44
**Self-rated academic performance level** (Missing = 18)		
Far above average/somewhat above average	591	27.26
Average	1116	51.48
Somewhat below average/far below average	461	21.26
**PHQ-9 score ranges**		
None, minimal, mild 0–9	1829	83.67
Moderate to severe 10–27	357	16.33

Personal stigma status, as well as the differences between male and female, only child and none-only child groups are shown in Table 2. The mean total score of the DSS-Personal scale was 13.71 (SD = 5.35), and males displayed significantly higher total scores than females (14.85 vs. 12.99, *P* < 0.0001). The most common stigmatizing attitudes toward depressed person were ‘I would not vote for a class cadre if I knew they had been depressed’ (31.13%), ‘Problem is a sign of personal weakness’ (30.9%) and ‘People with this problem are unpredictable’ (30.72%). Males were more likely to agree that ‘I would not vote for a class cadre if I knew they had been depressed’ than females (37.25 vs. 27.17%, *P* < 0.0001). The proportion of male respondents who had a belief in ‘I would not make friends with him if I knew he had been depressed’ (23.11 vs. 12.13%, *P* < 0.0001) and ‘Avoid people with this problem’ (13.89 vs. 5.62%, *P* < 0.0001) was twice that of females. Similarly, compared with non-only child, only child held a more stigmatizing attitude toward depressed person in many respects including ‘vote for a class cadre’ (39.13 vs. 27.32%, *P* < 0.0001) and ‘make friends’ (22.16 vs. 13.66%, *P* < 0.0001).

**Table 2.** Percentage of participants who ‘agree’ or ‘strongly agree’ with statements about their attitudes toward the person in the vignette.

Statement about personal belief (DSS)	Total (*N* = 2,186)		Gender					Whether only-child				
	*n*	%	Male (*n* = 838)		Female (*n* = 1,338)		*P*-value	Yes (*n* = 716)		No (*n* = 1,466)		*P*-value
			*n*	% (95% CI)	*n*	% (95% CI)		*n*	% (95% CI)	*n*	% (95% CI)	
1. The person could snap out of the problem (Missing = 5)	105	4.81	50	5.99 (4.38, 7.60)	54	4.04 (2.99, 5.10)	**0.0389**	23	3.23 (1.93, 4.52)	81	5.53 (4.36, 6.70)	**0.0179**
2. Problem is a sign of personal weakness (Missing = 5)	674	30.9	254	30.42 (27.30, 33.54)	417	31.21 (28.73, 33.70)	0.6971	210	29.45 (26.11, 32.8)	463	31.63 (29.24, 34.01)	0.3033
3. Problem is not a real medical illness (Missing = 5)	291	13.34	121	14.49 (12.10, 16.88)	169	12.65 (10.87, 14.43)	0.2199	90	12.62 (10.19, 15.06)	200	13.66 (11.90, 15.42)	0.5034
4. People with this problem are dangerous (Missing = 5)	449	20.59	179	21.44 (18.65, 24.22)	269	20.13 (17.98, 22.29)	0.4657	147	20.62 (17.65, 23.59)	301	20.56 (18.49, 22.63)	0.9754
5. Avoid people with this problem (Missing = 7)	192	8.81	116	13.89 (11.55, 16.24)	75	5.62 (4.39, 6.86)	**<.0001**	82	11.53 (9.19, 13.88)	109	7.45 (6.10, 8.79)	**0.0016**
6. People with this problem are unpredictable (Missing = 5)	670	30.72	232	27.78 (24.75, 30.82)	435	32.56 (30.05, 35.07)	**0.0190**	225	31.56 (28.15, 34.97)	444	30.33 (27.97, 32.68)	0.5597
7. If I had this problem, I would not tell anyone (Missing = 5)	377	17.29	150	17.96 (15.36, 20.57)	224	16.77 (14.76, 18.77)	0.4722	121	16.97 (14.22, 19.73)	256	17.49 (15.54, 19.43)	0.7653
8. I would not make friends with him if I knew he had been depressed (Missing = 5)	358	16.41	193	23.11 (20.25, 25.97)	162	12.13 (10.38, 13.88)	**<.0001**	158	22.16 (19.11, 25.21)	200	13.66 (11.90, 15.42)	**<.0001**
9. I would not vote for a class cadre if I knew they had been depressed (Missing = 5)	679	31.13	311	37.25 (33.97, 40.52)	363	27.17 (24.79, 29.56)	**<.0001**	279	39.13 (35.55, 42.71)	400	27.32 (25.04, 29.61)	**<.0001**
DSS total score (mean ± SD)	13.71 ± 5.35		14.85 ± 5.41		12.99 ± 5.17		**<.0001**	14.38 ± 5.22		13.38 ± 5.38		**<.0001**

Note: Boldface indicates statistical significance (P < 0.05).

Table 3 presents the results of three multivariate linear regression analysis. Higher personal stigma was significantly associated with more severe depressive symptoms in female group (Model 1) (*ß* = 1.75, *P* < 0.0001). For male group (Model 2), being only child (*ß* = 1.01, *P* = 0.0083) and moderate-to-severe depression were significantly related to personal stigma. In addition, Respondents who rated themselves as ‘competitive’ (*ß* = 1.29, *P* = 0.0088) or ‘Not at all/Somewhat competitive’ (*ß* = 1.04, *P* = 0.0381) showed more stigmatizing attitudes than ‘extremely/very competitive’ students. Significant interactions were found between gender and self-rated academic core competitiveness in Model 3 (Competitive: *P* = 0.0024, Not at all/Somewhat competitive: *P* = 0.0367).

**Table 3.** Multivariate linear regression analysis of factors associated with stigma.

Variables	Model 1: Female (*n* = 1,338)					Model 2: Male (*n* = 838)				Model 3: *P*-value interaction between gender and variables
	Estimate	SE	*t* Value	*P*-value		Estimate	SE	*t* Value	*P*-value	
Constant	12.97	2.11	6.16	**<.0001**		17.63	2.70	6.52	**<.0001**	**<.0001**
**Age**	0.00	0.09	−0.02	0.9806		−0.18	0.11	−1.65	0.0987	0.1964
**Whether only-child** (Ref = No)										
Yes	0.20	0.34	0.61	0.5420		1.01	0.38	2.65	**0.0083**	0.1098
**Self-rated academic core competitiveness** (Ref = Extremely/very competitive)										
Competitive	−0.46	0.31	−1.46	0.1451		1.29	0.49	2.63	**0.0088**	**0.0024**
Not at all/Somewhat competitive	−0.32	0.42	−0.76	0.4503		1.04	0.50	2.08	**0.0381**	**0.0367**
**Self-rated academic performance level** (Ref = Far above average/somewhat above average)										
Average	0.18	0.33	0.54	0.5913		0.57	0.46	1.23	0.2177	0.4876
Somewhat below average/far below average	−0.27	0.41	−0.65	0.5136		−0.78	0.56	−1.40	0.1612	0.4530
**Depression (PHQ-9)** (Ref = None, minimal, mild 0–9)										
Moderate to severe 10–27	1.75	0.38	4.60	**<.0001**		1.12	0.52	2.17	**0.0302**	0.3190

Note: Boldface indicates statistical significance (P < 0.05).

## Discussion

In the present study, the mean DSS-Personal scale among Chinese medical students was 13.71 ± 5.35, which was higher than previously published scores for Chilean adolescents (11.9 ± 4.8) [31] and for community adults in Portugal (12.71 ± 5.52) [32]. This finding suggests that the level of personal depression stigma among Chinese medical students is relatively high. In addition, above 30% respondents would not ‘vote for a class cadre’ for depressed person, regarded depression as ‘a sign of personal weakness’ and considered depressed people to be ‘unpredictable’. These findings manifested that a considerable number of students still lack understanding of the causes and clinical symptoms of depression, which needs to be reinforced in our school health education.

In this study, male students showed more stigmatizing attitudes toward people with depression than female, which is consistent with previous studies [22,33,34]. An interesting finding was that more male students were unwilling to interact or cooperate closely with people with depression than female students. To be specific, male students were more likely to agree that ‘I would not vote for a class cadre if I knew they had been depressed’ compared with female students; males were more unwilling to make friends with people with depression compared with female students; male students also were more likely to ‘avoid people with this problem’ compared with female students. From the perspective of social psychology, females are more willing to disclose themselves than males [35]. Once they have pressure or negative emotions, women are more willing to seek emotional support and talk to others. In addition, females have more ways to vent their emotions and are easier to digest negative emotions. There is an old Chinese saying that ‘A man’s tears are not lightly shed’. Men tend not to talk easily with others about their worries and do not reach out for help when faced with unfavorable mental health. A national online survey of UK medical students found that females felt less stigma than males in almost all conditions, including depression, psychotic symptoms, and long-term unexplained abdominal diseases [24]. Moreover, a study conducted among Tunisian students suggested that females had more knowledge about mental illness [36], and it has been established that knowledge has a direct negative correlation with depression stigma [37]. Studies from several universities in Hunan Province, China, showed that there was no gender difference in depression stigma scores, which differs from the results of this study [21]. This may be attributed to the fact that the subjects in the above study were non-medical students in China.

An interesting finding is that being an only child scored higher on the DSS-Personal scale than the none-only child. The only children are the product of a particular era and policy in China. Most of the only children are ‘little princesses’ and ‘little princes’ in the family. As the center of attention for several generations, only children are often over protected and develop a character of self-centered and dependence. However, they have to face the pressure of academic requirements and job-hunting alone in college. The huge contrast makes the only child more vulnerable, which leads to mental health problems. Moreover, the only child not only enjoy all the favors of his parents and elders from a young age but also carries the expectations of the whole family, so greater psychological pressure from parental expectations is inevitable. Especially, our research showed being an only child showed significantly associated with more stigmatization in terms of making friends and voting for a class cadre. The result indicates that only children usually display less trust toward depressed person. Results of a study examining the only-child effect by measuring brain activity and brain imaging in online trust games showed that only-child group showed a weaker interpersonal synchronization in the medial prefrontal cortex (mPFC) [38], which can reflect the decision to trust [39]. This research may contribute to our finding neurologically. Therefore, the psychological problems of the only children may have certain particularities, and the research and targeted guidance on the psychological health of the only children should be strengthened in the future.

In multivariate linear regression analysis, more severe depression has shown a significant association with more personal stigma, which was parallel with previous studies. A Chinese survey demonstrated that depressive symptom was positively correlated to personal stigma toward depression [28]. A study of US medical students showed that students with higher scores on the PHQ-9 scale were at greater risk of self-stigma [16]. Another study from Gujarat reported that 68.2% of medical students with moderate-to-severe depression felt embarrassed or ashamed if they were depressed, compared to 31.5% of students with no to mild depression [40]. Furthermore, our study first demonstrated that the self-rated academic core competitiveness was negatively associated with personal stigma for male group. Furthermore, our study first demonstrated the self-rated academic core competitiveness was negatively associated with personal stigma for male group. The self-rated academic core competitiveness is an individual’s subjective evaluation of his academic advantages, which is formed based on the individual’s subjective feelings and objective information about his own learning ability. Individuals with higher self-rated academic core competitiveness tend to have stronger psychological capital, and thus have lower depression stigma. Our finding indicated that academic core competitiveness is needed to strengthen efforts at combating depression stigma among male college students. More importantly, this study confirms that there are gender differences in depression stigma. Therefore, there is a need for gender-differentiated mental health education among Chinese medical students [21,31].

Limitations exist. First, the analysis of cross-sectional data limited our ability to establish causality between independent and dependent variables. Therefore, it is necessary to conduct longitudinal study to validate the current findings. Second, our sample was confined to medical students in a southernmost province, which may limit the generalization of the findings to other areas of China or other countries.

## Conclusion

To our knowledge, the present study is the first to explore gender differences in predictors of depression stigma among Chinese medical students. The present study shows that personal depression stigma was common in Chinese medical students, especially male students. And more males were unwilling to interact or cooperate closely with depressed people than females. For male group, higher personal stigma was predicted by being only child, depression symptom, and lower self-rated academic core competitiveness, while for female group, personal stigma was only associated with depression symptom. In particular, the effect of lower self-rated academic core competitiveness on stigma was significantly different between male and female groups. Although most universities in China offer courses related to mental health education, the current education neglects knowledge on how to reduce mental health stigma. Therefore, effective measures must be taken to reduce mental health stigma and gender differences need to be taken into account in this process.
